# Discovering the Clinical and Prognostic Role of Pan-Immune-Inflammation Values on Oral Cavity Squamous Cell Carcinoma

**DOI:** 10.3390/cancers15010322

**Published:** 2023-01-03

**Authors:** Chia-Chi Yeh, Huang-Kai Kao, Yenlin Huang, Tsung-You Tsai, Chi-Kuang Young, Shao-Yu Hung, Chuieng-Yi Lu, Kai-Ping Chang

**Affiliations:** 1 Department of Otolaryngology-Head and Neck Surgery, Chang Gung Memorial Hospital at Linkou Branch, Taoyuan 333423, Taiwan; 2 Department of Plastic and Reconstructive Surgery, Chang Gung Memorial Hospital at Linkou Branch, Taoyuan 333423, Taiwan; 3 College of Medicine, Chang Gung University, Taoyuan 333323, Taiwan; 4 Department of Anatomic Pathology, Chang Gung Memorial Hospital at Linkou Branch, Taoyuan 333423, Taiwan; 5 Institute of Stem Cell and Translation Cancer Research, Chang Gung Memorial Hospital at Linkou Branch, Taoyuan 333423, Taiwan; 6 School of Medicine, National Tsing-Hua University, Hsinchu 300044, Taiwan; 7 Molecular Medicine Research Center, Chang Gung University, Taoyuan 333323, Taiwan

**Keywords:** oral cavity, squamous cell carcinoma, pan-immune-inflammation value, OSCC, head and neck

## Abstract

**Simple Summary:**

A newly introduced pan-immune-inflammation value (PIV) was evaluated for its prognostic role in many cancers but not oral cavity squamous cell carcinoma (OSCC). We retrospectively reviewed 853 OSCC patients from 2005 to 2017, and the optimal preoperative PIV was determined by a receiver operating characteristic curve. Significant differences were observed for pT status, pN status, overall pathological status, extranodal extension, cell differentiation, depth of invasion, and perineural invasion between higher and lower PIV patients. Kaplan-Meier and univariate regression analyses indicated that higher PIV was associated with worse overall survival, disease-free survival, locoregional recurrence-free survival, and distant metastasis-free survival. Multivariate analyses adjusted by various factors further demonstrated that PIV was an independent prognostic factor for overall and distant metastasis-free survival. Overall, a higher PIV level was associated with clinicopathological factors in OSCC patients and could be used to predict worse outcomes, especially overall and distant metastasis-free survival.

**Abstract:**

A newly introduced pan-immune-inflammation value (PIV) was not evaluated for its role in oral cavity squamous cell carcinoma (OSCC). In this study, the PIV was calculated with the following equation (neutrophil count × platelet count × monocyte count)/lymphocyte count from the results of the automated hematology analyzers in 853 OSCC patients from 2005 to 2017. The optimal cutoff for the preoperative PIV was 268, as determined by a receiver operating characteristic curve. Significant differences were observed for alcohol consumption, smoking, pT status, pN status, overall pathological status, extranodal extension, cell differentiation, depth of invasion, and perineural invasion between higher and lower PIV patients (all *p* values < 0.05). Kaplan-Meier and univariate regression analyses indicated that higher PIV was associated with worse overall survival, disease-free survival, locoregional recurrence-free survival, and distant metastasis-free survival (all *p* values < 0.001). Multivariate analyses adjusted by various factors further demonstrated that PIV was an independent prognostic factor for overall and distant metastasis-free survival (*p* = 0.027, HR: 1.281 and *p* = 0.031, HR: 1.274, respectively). In conclusion, a higher PIV level was associated with poor clinicopathological factors in OSCC patients and could be used to predict poor posttreatment outcomes, especially for overall and distant metastasis-free survival.

## 1. Introduction

Head and neck cancer is an aggressive disease that is life-threatening without adequate treatment. It is also the seventh most common cancer in the world, with an annual incidence of approximately 700,000 and a mortality rate estimated at 350,000 in 2018. Among all head and neck regions, the oral cavity is the most frequent location, and the dominant histologic type is squamous cell carcinoma [1,2]. The tumor-node-metastasis staging system has been used for general guidelines and outcome evaluation over four decades to guide head and neck cancer treatment. Currently, the predominant therapeutic strategies for oral cavity squamous cell carcinoma (OSCC) usually involve primary ablative surgery and adjuvant therapy, including radiotherapy or chemoradiotherapy, for patients with worse prognostic factors. The prognostication of some patients is still challenging. Overall, the five-year relative survival of OSCC patients was only 49%, and many experienced locoregional recurrence or distant metastasis [2,3].

Increasing evidence has indicated that cancer progression and advancement are also correlated with systemic inflammatory responses [4,5,6,7,8]. Past studies have combined different clinical biomarkers to enhance prognostic value, including systemic inflammation scores. In accordance with our previous reports, a higher systemic inflammation score and other related measurements were associated with many poor prognostic factors and poor disease-free survival after OSCC treatment [9,10,11]. In 2020, Fuca et al. introduced a new biomarker, pan-immune-inflammation value (PIV), which has been proven to be a comprehensive predictor of survival outcomes with better performance than other biomarkers, such as the neutrophil-lymphocyte ratio and platelet-lymphocyte ratio, in patients with metastatic colorectal cancer [12]. In addition, more recent studies showed that PIV was a more accurate marker for predicting patient outcomes in solid organ malignancies [12,13,14,15,16,17].

Although PIV has been studied and reported in many cancers, its association with clinicopathological manifestations and its prognostic value in patients with head and neck cancer have not been elucidated. Therefore, the current study aimed to investigate the association between PIV and the clinicopathological characteristics of OSCC. Furthermore, the predictive value of OSCC posttreatment outcomes by PIV was evaluated by univariate and multivariate survival analyses.

## 2. Materials and Methods

### 2.1. Patients

In this study, the clinicopathological data of a cohort of OSCC patients from 2005 to 2017 were retrospectively reviewed. Patients who visited the otolaryngology clinic for OSCC treatment were consecutively recruited. Patients with the following conditions were excluded: a previous history of any malignancy, known distant metastasis or second primary cancer diagnosed before treatment, or a history of neoadjuvant radiation or chemotherapy. Informed consent, approved by the Institutional Review Board of Chang Gung Memorial Hospital (Approval number: 201305685A3), was obtained. The patients were defined as betel nut chewers if they chewed 2 or more betel nuts daily for at least 1 year, as cigarette smokers if they smoked every day for at least 1 year, and as alcohol drinkers if they consumed an alcoholic beverage 1 or more times per week for at least 6 months. The PIV was calculated with the following equation (neutrophil count (10^3^/mL) × platelet count (10^3^/mL) × monocyte count (10^3^/mL))/lymphocyte count (10^3^/mL) from the results of the automated hematology analyzers [12].

All OSCC patients were previously untreated and received surgery as the primary treatment modality. A thorough review of their medical history, physical examination, laboratory data, chest radiographs, imaging studies (including computed tomography or magnetic resonance imaging), liver ultrasonography, positron emission tomography or bone scans was conducted before the treatment was initiated. An age of more than 65 was considered as the elderly population [10,11]. Primary tumors were excised with adequate margins using intraoperative frozen section controls transorally or via lip splitting, and neck dissections were simultaneously performed at level I–III (for clinical N0 neck disease) or level I–V (for clinical N+ disease). According to the defect size, primary closure or flap reconstruction was performed immediately after tumor ablation. The adequacy of the resection margin was assessed with intraoperative frozen sections. Pathological staging was determined according to the 8th edition of the American Joint Committee on Cancer staging criteria [18,19]. Adjuvant radiotherapy or chemoradiotherapy was administered after the tumor board discussion, mainly according to the National Comprehensive Cancer Network guidelines. Generally, postoperative radiation therapy was considered if close (<5 mm) margins, perineural invasion, bone invasion, and advanced T stage (T3–T4) were present. Adjuvant chemoradiotherapy was considered if patients presented with positive margins, extranodal extension (ENE), or pathologic multiple nodal metastases. The prescribed dose was 2 Gy per fraction per day. The total radiation dose for patients was 60–75 Gy. The patients were followed up in the outpatient clinic every 2–3 months in the first year, every 3–4 months during the second and third years, and every 6 months thereafter.

### 2.2. Statistical Analysis

All statistical analyses were performed using SAS software (version 9.4; SAS Institute, Cary, NC, USA). Chi-square and Wilcoxon tests were used to test the differences in the clinicopathological features between the high and low PIV patients. The area under the curve was calculated using receiver operating characteristic analysis, and the optimal cutoff PIV value was chosen by using Youden’s J statistic to select the best cutoff value (maximum Youden’s index). Survival rates were demonstrated using Kaplan–Meier plots and were examined with the log-rank test. Furthermore, the association of the variables and survival was further analyzed with univariate and multivariate Cox regression models. All patients were followed up until August 2021 or their demise. All *p* values were two-sided, and a *p* value less than 0.05 indicated statistical significance.

## 3. Results

### 3.1. Patient Characteristics and Clinicopathological Data

In the current study, 853 OSCC patients were enrolled from 2005 to 2017 with a mean age of 53.5 years, and 780 (91.4%) were men; 83.9% were smokers, 68.3% were alcohol consumers, and 81.9% were betel nut chewers. The period between the blood tests and surgery was 4.9 ± 2.8 days. The detailed clinicopathological characteristics of the OSCC patients are shown in Table 1.

### 3.2. Association between PIV Groups and Patient Clinicopathological Characteristics

**Figure 1:**
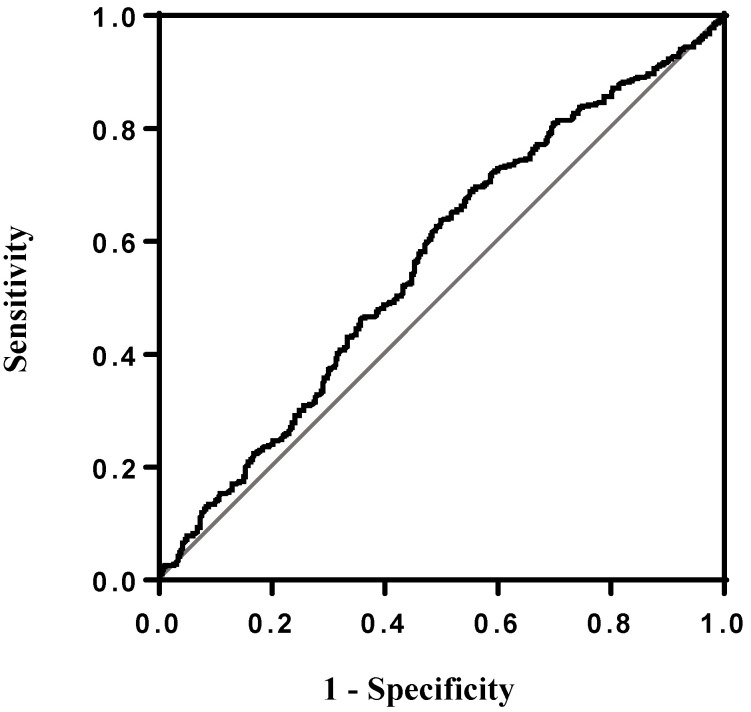
Receiver operating characteristic (ROC) curve showing the predictive efficacy of overall. survival between higher (≥268) and lower (<268) PIV patients. The area under curve of PIV in the ROC curve was 0.566.

Receiver operating characteristic curve analysis was constructed (Figure 1), and Youden’s J statistic was used to stratify OSCC patients into high PIV and low PIV groups. The best cutoff PIV value (maximum Youden’s index) was 268 and the clinicopathological factors are compared in Table 2. Significant differences in sex, alcohol consumption, betel nut chewing, smoking, pT status, pN status, overall pathological status, extranodal extension, cell differentiation, lymphovascular invasion, depth of invasion, and perineural invasion were observed between higher (≥268) and lower (<268) PIV patients (*p* values < 0.001, =0.005, <0.001, =0.005, <0.001, <0.001, <0.001, <0.001, =0.006, =0.024, <0.001, and <0.001, respectively).

### 3.3. Association between PIV Groups and Survival Status in OSCC Patients

**Figure 2:**
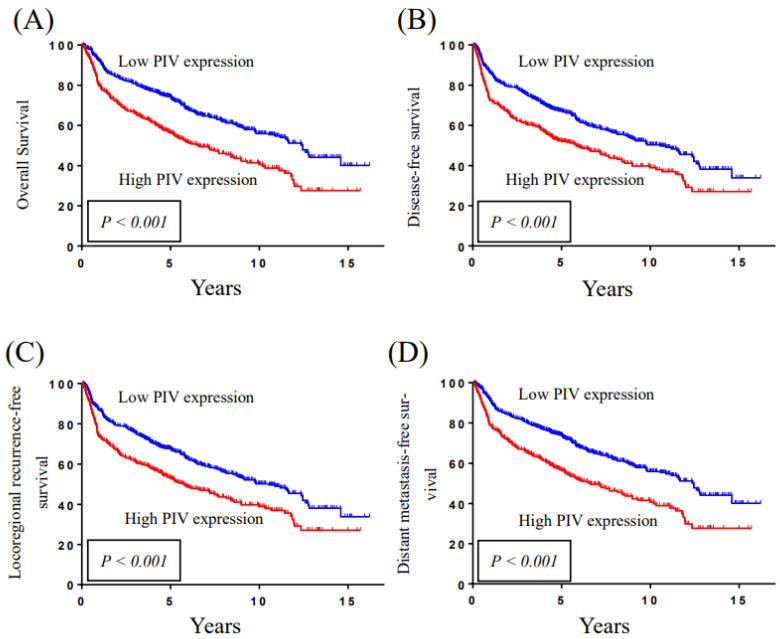
Kaplan-Meier survival curve demonstrates that the 5-year (**A**) overall survival, (**B**) disease-free survival, (**C**) locoregional recurrence-free survival, and (**D**) distant metastasis-free survival rates for patient subgroups stratified by PIV levels using 268 as the cutoff values were 74.1% vs. 56.4%, 67.1% vs. 52.3%, 67.6% vs. 53.1%, and 73.9% vs. 56.5%, respectively (*p* < 0.001, <0.001, <0.001, and <0.001). The *p* values were calculated with the log-rank test.

The Kaplan–Meier plots in Figure 1 illustrate the comparison of survival outcomes between higher and lower PIV groups. Using 268 as a cutoff value, higher PIV patients were significantly associated with poorer overall survival, disease-free survival, locoregional recurrence-free survival, and distant metastasis-free survival. According to the Kaplan-Meier survival curves, the 5-year overall survival, disease-free survival, locoregional recurrence-free survival, and distant metastasis-free survival rates for patient subgroups stratified by PIV levels using 268 as the cut-off value were 74.1% vs. 56.4%, 67.1% vs. 52.3%, 67.6% vs. 53.1%, and 73.9% vs. 56.5%, respectively (*p* < 0.001, <0.001, <0.001, and <0.001; Figure 2). The *p* values were calculated using a log-rank test.

By the Cox proportional hazard model, univariate analysis revealed that age, overall pathological stage (stage III–IV vs. I–II), surgical margin (< vs. ≥5 mm), extranodal extension (positive vs. negative), cell differentiation (poor differentiation vs. good and moderate differentiation), perineural invasion (positive vs. negative), depth of invasion (<10 mm vs. ≥10 mm), lymphovascular invasion (positive vs. negative), adjuvant therapy (without vs. with radiotherapy or chemoradiotherapy), and PIV were significantly associated with overall survival, disease-free survival, locoregional recurrence-free survival, and distant metastasis-free survival (Table 3).

Furthermore, after adjusting for age, sex, overall pathological stage, extranodal extension, perineural invasion, lymphovascular invasion (positive vs. negative), surgical margin, histological differentiation, depth of invasion, and adjuvant therapy (without vs. with radiotherapy or chemoradiotherapy), PIV was found to be an independent prognostic factor for overall survival and distant metastasis-free survival (*p* = 0.027 and 0.031, respectively; Table 4).

## 4. Discussion

Increasing evidence has indicated that cancer progression and advancement are associated with systemic inflammatory responses. Traditionally, inflammation in the body can be detected in blood analysis through some clinical biomarkers, such as neutrophils, lymphocytes, platelets, and monocytes [4,5,6,7,8]. In addition, their ratios can be used to predict the patient’s outcome, and past studies have tried to combine different clinical biomarkers to improve their prognostic value, including the neutrophil/lymphocyte ratio (NLR), platelet/lymphocyte ratio (PLR), and systemic inflammation score [11]. Increased neutrophil count and/or decreased lymphocyte count may suppress lymphokine-activated killer cells, and lymphocytopenia may be considered a marker of generalized immune depression status, which affects patient survival [20,21]. Therefore, a higher pretreatment NLR level might be related to favorable conditions for tumor growth, aggressiveness, and recurrence [22]. There are also reports that platelets reduce the cytolytic activity of natural killer cells and thus influence patients’ immunity [21]. A higher PLR level also showed a similar impact to NLR on patient outcomes. Studies have shown that the PLR is better than the NLR in predicting disease-free survival and overall survival. It can be considered an independent prognostic indicator in OSCC patients [21,22,23].

In addition to NLR and PLR, Chang et al. [24] established a prognostic evaluation score, the systemic inflammation score, which was used to predict the postoperative outcome of patients with clear-cell renal cell carcinoma. The score was calculated by combining the serum albumin level and the lymphocyte-to-monocyte ratio. In our clinical experience, a higher systemic inflammation score was significantly associated with many poor clinicopathological features and was also an independent prognostic factor for disease-free and overall survival of patients with OSCC [11].

The PIV, which integrates circulating platelets, monocytes, neutrophils, and lymphocytes, is a novel clinical biomarker that reflects the systemic immune-inflammation response. It has also been shown to precisely predict the outcomes of patients with colorectal, breast, small-cell lung, prostate cancers and malignant melanomas [10,12,13,15,17,25,26,27,28,29,30]. For instance, high PIV levels were reported to be associated with worse clinical outcomes in patients with small-cell lung cancer [14], and a lower PIV was also considered a biomarker indicating a good prognosis in metastatic colorectal cancer [12]. Generally, higher PIV levels indicated a significantly increased risk of death and disease progression than lower PIV levels. This result was predictable because the PIV level should be positively correlated with the NLR and PLR. Recently, Ligorio [13] reported that PIV showed a better association with survival and outperformed NLR and PLR in predicting survival in patients with human epidermal growth factor receptor 2-positive advanced breast cancer. PIV might be a more comprehensive and reliable clinical biomarker to predict posttreatment outcomes.

However, the prognostic role of the pretreatment PIV level is not well established for OSCC thus far. Therefore, we designed the current retrospective study and analyzed the clinicopathological characteristics, treatment outcomes and prognosis of 853 OSCC patients. To the best of our knowledge, this study is the first report to evaluate the role of PIV levels in OSCC patients or even any patients with head and neck cancers.

To investigate the relationship between the PIV levels and the clinicopathological characteristics of OSCC patients, we also elucidated the posttreatment outcomes by performing univariate and multivariate survival analyses. For the objective stratification of PIV levels, we performed receiver operating characteristic analysis, and the optimal cutoff PIV value was chosen by using Youden’s J statistic to select the best cutoff value (maximum Youden’s index). Therefore, a subjective cutoff value could be found and further used to define the higher (≥268) and the lower PIV level groups. In the current study, higher PIV levels were associated with alcohol consumption, smoking, and betel nut chewing. These results are compatible with the findings of some previous reports demonstrating that alcohol, cigarettes, and betel nuts are all carcinogens and that they could cause chronic inflammation and induce further tumor progression or the occurrence of second primary malignancies [8,31,32,33]. A higher PIV level was also associated with advanced pathological features (including pT status, pN status, overall pathological status, extranodal extension, cell differentiation, depth of invasion, and perineural invasion) in OSCC. These pathological features all reflect the aggressiveness of OSCC tumors. Higher PIV levels reflect high neutrophil levels, high platelet counts, high monocyte levels, or relatively low lymphocyte levels. Neutrophils can induce tumor angiogenesis and invasion through the production of cytokines such as vascular endothelial growth factor and interleukin-8 and the production of matrix metalloproteinase-9, and platelets can facilitate tumor angiogenesis through increased microvascular permeability [22]. Tumor metastasis could also be induced by monocytes through extracellular matrix remodeling and by platelets through binding to a specific adhesion molecule [21,22,23]. On the other hand, relative lymphocytopenia in the circulation or microenvironment is also a sign of immune depression and may indicate that the patients’ immunity might be insufficient to confront tumor progression and metastasis [20,21]. Taken together, higher PIV levels may be related to tumor metastasis, invasion and progression in the microenvironment, and be associated with nodal metastasis, extranodal extension, perineural invasion, and higher pT status, as noted in the current study.

According to the results of our study, PIV was shown to be a feasible and easily calculated index that can be used in any clinical setting. Laboratory tests of whole blood count would be performed routinely for every patient before treatment, and the data involved in the equation of the PIV level are acquired very easily and straightforwardly without further processing or conversion. Most importantly, the PIV provides an easy way to provide significant information related to the patients’ general condition and it may be useful for making better preoperative assessments and individualized treatment decisions.

## 5. Conclusions

The current study identified a convenient marker that could provide useful information related to the patients’ immune condition during OSCC treatment. The association between higher PIV levels and many poor clinicopathological factors in OSCC patients, such as pT status, pN status, overall pathological status, extranodal extension, cell differentiation, depth of invasion, and perineural invasion, was demonstrated. Furthermore, the OSCC patients with higher PIV levels had worse overall, disease-free, locoregional recurrence-free, and distant metastasis-free survival in univariate analyses. After adjusting for multiple clinicopathological features by multivariate analyses, PIV levels were shown to be an independent prognostic factor for overall survival and distant metastasis-free survival and thus could also be used to predict survival outcomes before treatment.

## Figures and Tables

**Table 1:** Baseline clinicopathological characteristics of patients with oral cavity squamous cell carcinoma (*n* = 853).

Variable	Characteristics
**Age (years)**	
<65	713 (83.6%)
≥65	140 (16.4%)
**Gender**	
Male	780 (91.4%)
Female	73 (8.6%)
**Personal Habits**	
Alcohol consumption	583 (68.3%)
Betel nut chewing	699 (81.9%)
Cigarettes smoking	716 (83.9%)
**Tumor site**	
Buccal mucosa	323 (37.9%)
Tongue	310 (36.3%)
Others	220 (25.8%)
**Overall stage**	
I	164 (19.2%)
II	189 (22.2%)
III	129 (15.1%)
IV	371 (43.5%)
**pT classification**	
T1	194 (22.7%)
T2	286 (33.5%)
T3	91 (10.7%)
T4	282 (33.1%)
**pN classification**	
N0	547 (64.1%)
N1	116 (13.6%)
N2	188 (22.0%)
N3	2 (0.3%)
**PNI**	303 (35.6%)
**ENE**	171 (20.1%)
**LVI**	64 (7.5%)
**DOI ≥ 10 mm**	427 (50.1%)
**Surgical margin**	
<5 mm	260 (30.5%)
≥5 mm	593 (69.5%)
**Adjuvant therapy**	
Absent	366 (42.9%)
Radiotherapy	164 (19.2%)
Chemoradiotherapy	323 (37.9%)
**Neutrophil (×10^3^ μL^−1^) ^§^**	5.5 ± 16.5
**Platelets (×10^3^ μL^−1^) ^§^**	258.6 ± 92.0
**Monocyte (×10^3^ μL^−1^) ^§^**	0.5 ± 0.2
**Lymphocyte (×10^3^ μL^−1^) ^§^**	2.0 ± 0.8
**PIV ^§^**	410.9 ± 1048.9

Abbreviations: PNI, perineural invasion; ENE, extranodal extension; LVI, lymphovascular invasion; DOI, depth of invasion; PIV: pan-immune-inflammation value. ^§^ Mean ± SD.

**Table 2:** Baseline clinicopathological characteristics according to the PIV.

	PIV		
Variable	<268 (*n* = 487)	≥268 (*n* = 366)	*p*-Value
**Age (years)**			
<65	400 (82.1%)	313 (85.5%)	0.186
≥65	87 (17.9%)	53 (14.5%)	
**Gender**			
Male	430 (88.3%)	350 (95.6%)	<0.001 *
Female	57 (11.7%)	16 (4.4%)	
**Alcohol consumption**			
No	173 (35.6%)	97 (26.6%)	0.005 *
Yes	313 (64.4%)	268 (73.4%)	
**Betel nut chewing**			
No	107 (22.0%)	47 (12.9%)	<0.001 *
Yes	379 (78.0%)	318 (87.1%)	
**Cigarettes smoking**			
(−)	93 (19.1%)	44 (12.1%)	0.005 *
(+)	393 (80.9%)	321 (87.9%)	
**pT classification**			
T1–T2	349 (71.7%)	131 (35.8%)	<0.001 *
T3–T4	138 (28.3%)	235 (64.2%)	
**pN classification**			
N0	345 (70.8%)	202 (55.2%)	<0.001 *
N1–N3	142 (29.2%)	164 (44.8%)	
**Overall Stage**			
I–II	268 (55.0%)	85 (23.2%)	<0.001 *
III–IV	219 (45.0%)	281 (76.8%)	
**ENE**			
Absent	421 (86.5%)	261 (71.3%)	<0.001 *
Present	66 (13.5%)	105 (28.7%)	
**Cell differentiation**			
W-D/M-D	443 (91.0%)	311 (85.0%)	0.006 *
P-D	44 (9.0%)	55 (15.0%)	
**LVI**			
Absent	459 (94.3%)	330 (90.2%)	0.024 *
Present	28 (5.7%)	36 (9.8%)	
**PNI**			
Absent	351 (72.1%)	198 (54.3%)	<0.001 *
Present	136 (27.9%)	167 (45.7%)	
**DOI**			
<10 mm	312 (64.1%)	114 (31.2%)	<0.001 *
≥10 mm	175 (35.9%)	252 (68.8%)	

Abbreviations: PNI, perineural invasion; ENE, extranodal extension; LVI, lymphovascular invasion; DOI, depth of invasion; PIV: pan-immune-inflammation value; W-D: well-differentiated, M-D: moderately differentiated, and P-D: poorly differentiated; * statistically significant.

**Table 3:** Univariate analysis of poor prognostic factors for OS, DFS, LRFS, and DMFS in OSCC patients.

	**OS**		**DFS**		**LRFS**		**DMFS**	
**Variable**	**HR (95% CI)**	***p*** **-Value**	**HR (95% CI)**	***p*** **-Value**	**HR (95% CI)**	***p*** **-Value**	**HR (95% CI)**	***p*** **-Value**
**Age (years)**								
<65	Reference		Reference		Reference		Reference	
≥65	1.622	<0.001 *	1.454	0.001 *	1.459	0.001 *	1.597	<0.001 *
	(1.275–2.064)		(1.149–1.839)		(1.154–1.846)		(1.254–2.035)	
**Gender**								
Female	Reference		Reference		Reference		Reference	
Male	0.833	0.275	0.773	0.102	0.773	0.102	0.832	0.269
	(0.601–1.156)		(0.567–1.053)		(0.567–1.053)		(0.599–1.154)	
**Overall Stage**								
I–II	Reference		Reference		Reference		Reference	
III–IV	2.614	<0.001 *	2.287	<0.001 *	2.282	<0.001 *	2.610	<0.001 *
	(2.084–3.279)		(1.852–2.824)		(1.848–2.818)		(2.080–3.274)	
**Surgical margin**								
<5 mm	Reference		Reference		Reference		Reference	
≥5 mm	1.352	0.005 *	1.282	0.016 *	1.279	0.017 *	1.353	0.004 *
	(1.096–1.668)		(1.047–1.571)		(1.045–1.567)		(1.096–1.670)	
**ENE**								
Absent	Reference		Reference		Reference		Reference	
Present	3.072	<0.001 *	2.865	<0.001 *	2.853	<0.001 *	3.096	<0.001 *
	(2.471–3.819)		(2.318–3.540)		(2.309–3.526)		(2.490–3.850)	
**Cell differentiation**								
W-D/M-D	Reference		Reference		Reference		Reference	
P-D	1.746	<0.001 *	1.745	<0.001 *	1.751	<0.001 *	1.758	<0.001 *
	(1.323–2.305)		(1.337–2.277)		(1.341–2.285)		(1.331–2.320)	
**LVI**								
Absent	Reference		Reference		Reference		Reference	
Present	1.721	0.001 *	1.631	0.003 *	1.608	0.004 *	1.731	0.001 *
	(1.231–2.406)		(1.177–2.260)		(1.161–2.228)		(1.238–2.420)	
**PNI**								
Absent	Reference		Reference		Reference		Reference	
Present	1.871	<0.001 *	1.718	<0.001 *	1.716	<0.001 *	1.860	<0.001 *
	(1.530–2.289)		(1.415–2.084)		(1.414–2.082)		(1.520–2.275)	
**DOI**								
<10 mm	Reference		Reference		Reference		Reference	
≥10 mm	2.309	<0.001 *	2.047	<0.001 *	2.041	<0.001 *	2.303	<0.001 *
	(1.877–2.841)		(1.682–2.490)		(1.678–2.484)		(1.871–2.834)	
**Adjuvant Tx**								
Without	Reference		Reference		Reference		Reference	
With	2.326	<0.001 *	2.199	<0.001 *	2.195	<0.001 *	2.321	<0.001 *
	(1.868–2.896)		(1.787–2.706)		(1.784–2.701)		(1.864–2.890)	
**PIV**								
<268	Reference		Reference		Reference		Reference	
≥268	1.723	<0.001 *	1.531	<0.001 *	1.521	<0.001 *	1.717	<0.001 *
	(1.410–2.105)		(1.264–1.854)		(1.255–1.842)		(1.405–2.099)	

Abbreviations: OS: overall survival; DFS: disease-free survival; LRFS: locoregional recurrence-free survival; DMFS: distance metastasis-free survival; HR: hazard ratio; CI: confidence interval; ENE, extranodal extension; W-D: well-differentiated, M-D: moderately differentiated, and P-D: poorly differentiated; LVI, lymphovascular invasion; PNI, perineural invasion; DOI, depth of invasion; Adjuvant tx: radiotherapy or chemoradiotherapy; PIV: pan-immune-inflammation value; * Statistically significant.

**Table 4:** Multivariate analysis of poor prognostic factors for OS, DFS, LRFS, and DMFS in OSCC patients.

	OS		DFS		LRFS		DMFS	
Variable	HR (95% CI)	*p*-Value	HR (95% CI)	*p*-Value	HR (95% CI)	*p*-Value	HR (95% CI)	*p*-Value
**Age (years)**								
<65	Reference		Reference		Reference		Reference	
≥65	1.023	<0.001 *	1.018	<0.001 *	1.018	<0.001 *	1.022	<0.001 *
	(1.014–1.032)		(1.009–1.027)		(1.010–1.027)		(1.013–1.031)	
**Gender**								
Female	Reference		Reference		Reference		Reference	
Male	0.823	0.257	0.780	0.126	0.783	0.132	0.824	0.260
	(0.588–1.153)		(0.567–1.073)		(0.569–1.077)		(0.589–1.154)	
**Overall Stage**								
I–II	Reference		Reference		Reference		Reference	
III–IV	1.460	0.025 *	1.292	0.111	1.290	0.114	1.459	0.026 *
	(1.047–2.036)		(0.943–1.770)		(0.941–1.769)		(1.046–2.034)	
**ENE**								
Absent	Reference		Reference		Reference		Reference	
Present	2.004	<0.001 *	1.961	<0.001 *	1.963	<0.001 *	2.024	<0.001 *
	(1.551–2.589)		(1.530–2.514)		(1.531–2.516)		(1.565–2.617)	
**Surgical margin**								
<5 mm	Reference		Reference		Reference		Reference	
≥5 mm	1.013$$%(0.816–1.256)	0.910	0.997$$%(0.810–1.227)	0.975	0.994$$%(0.807–1.224)	0.953	1.013$$%(0.816–1.257)	0.904
**DOI**								
<10 mm	Reference		Reference		Reference		Reference	
≥10 mm	1.468	0.004 *	1.359	0.017 *	1.370	0.014 *	1.467	0.004 *
	(1.126–1.915)		(1.055–1.750)		(1.064–1.764)		(1.124–1.913)	
**Cell Differentiation**								
W-D/M-D	Reference		Reference		Reference		Reference	
P-D	1.117	0.456	1.169	0.275	1.184	0.237	1.126	0.426
	(0.834–1.497)		(0.883–1.546)		(0.895–1.566)		(0.841–1.509)	
**PNI**								
Absent	Reference		Reference		Reference		Reference	
Present	1.160	0.221	1.092	0.452	1.088	0.468	1.144	0.268
	(0.914–1.472)		(0.868–1.373)		(0.865–1.369)		(0.901–1.453)	
**LVI**								
Absent	Reference		Reference		Reference		Reference	
Present	0.873	0.458	0.893	0.525	0.881	0.477	0.880	0.485
	(0.609–1.251)		(0.630–1.266)		(0.622–1.249)		(0.614–1.261)	
**Adjuvant tx**								
Without	Reference		Reference		Reference		Reference	
With	1.055	0.751	1.174	0.326	1.170	0.337	1.056	0.749
	(0.757–1.472)		(0.852–1.617)		(0.849–1.613)		(0.757–1.472)	
**PIV**								
<268	Reference		Reference		Reference		Reference	
≥268	1.281	0.027 *	1.165	0.157	1.159	0.170	1.274	0.031 *
	(1.027–1.596)		(0.943–1.438)		(0.939–1.432)		(1.022–1.588)	

Abbreviations: OS: overall survival; DFS: disease-free survival; LRFS: locoregional recurrence-free survival; DMFS: distance metastasis-free survival; HR: hazard ratio; CI: confidence interval; ENE, extranodal extension; DOI, depth of invasion; W-D: well-differentiated, M-D: moderately differentiated, and P-D: poorly differentiated; PNI, perineural invasion; LVI, lymphovascular invasion; Adjuvant tx: radiotherapy or chemoradiotherapy; PIV: pan-immune-inflammation value; * Statistically significant.

## Data Availability

The data presented in this study are available on request from the corresponding authors. The data are not publicly available due to ethical considerations.
